# Reproducibility and Validity of the Myotest for Measuring Step Frequency and Ground Contact Time in Recreational Runners

**DOI:** 10.1515/hukin-2015-0003

**Published:** 2015-04-07

**Authors:** Vincent Gouttebarge, Robin Wolfard, Nouschka Griek, Cornelis J. de Ruiter, Julitta S. Boschman, Jaap H. van Dieën

**Affiliations:** 1 Department of Orthopaedic Surgery, Academic Medical Center, Amsterdam, The Netherlands.; 2 MOVE Research Institute Amsterdam, Faculty of Human Movement Sciences, VU University Amsterdam, Amsterdam, The Netherlands.; 3 Vintta | Research and Consultancy for Sport Health, Almere, The Netherlands.; 4 King Abdulaziz University, Jeddah, Saudi Arabia.

**Keywords:** agreement, concurrent validity, step frequency, ground contact time

## Abstract

The purpose of this study was to assess the reproducibility (test-retest reliability and agreement) and concurrent validity of the Myotest for measuring step frequency (SF) and ground contact time (GCT) in recreational runners. Based on a within-subjects design (test and retest), SF and GCT of 14 participants (11 males, 3 females) were measured at three different running speeds with the Myotest during two test sessions. SF and GCT were also assessed with a foot-mounted accelerometer (Gold Standard, previously validated by comparing to force plate data) during the first test session. Levels of test-retest reliability and concurrent validity were expressed with intraclass correlation coefficients (ICC), agreement with standard errors of measurement (SEM). For SF, test-retest reliability (ICC’s > 0.75) and agreement of the Myotest were considered as good at all running speeds. For GCT, test-retest reliability was found to be moderate at a running speed of 14 km/h and poor at speeds of 10 and 12 km/h (ICC < 0.50). Agreement of the Myotest for GCT at all three running speeds was considered not acceptable given the SEM’s calculated. Concurrent validity of the Myotest with the foot-mounted accelerometer (Gold Standard) at all three running speeds was found to be good for SF (ICC’s > 0.75) and moderate for GCT (0.50 < ICC’s < 0.75). The conclusion of our study is that estimates obtained with the Myotest are reproducible and valid for SF but not for GCT.

## Introduction

As a consequence of its practicality and positive effects for physical health and mental well-being, running has in the past years become one of the most popular forms of physical activity (Thompson Coon et al., 2011; Williams, 2012a; Williams, 2012b). The total number of recreational runners has increased by 18% from 2007 to 2008 in the United States (Running-U.S.A, 2012), while the running population doubled within the latest decade in the Netherlands (van Bottenburg, 2009).

Next to its beneficial health effects, running is also associated with negative effects, runners being at high risk of musculoskeletal injuries (Hespanhol Junior et al., 2011; Lopes et al., 2012). A new acute musculoskeletal injury occurs in one out of five runners during a marathon, with injury lasting longer than 3 months in 25% of them (van Middelkoop et al., 2008). Known risk factors for running injuries are diverse, among which gender, high body mass index, history of previous running injuries, muscle functions and weekly training distance and frequency are the most important ones (van Middelkoop et al., 2008; Buist et al., 2010; Lopes et al., 2012; Moen et al., 2012). Lately, running technique elements have gained attention as risk factors for musculoskeletal injuries. Several authors have suggested that many running injuries might derive from poor running technique and that alterations in running technique elements, such as step frequency, stride length, vertical oscillation, ground contact time or foot strike pattern, decrease the biomechanical load on lower extremities, which might prevent the occurrence of musculoskeletal injuries (Collier, 2011; Lieberman, 2012; Rixe et al., 2012; Boschman and Gouttebarge, 2013). Consequently, measuring and monitoring running technique elements such as step frequency (SF) and ground contact time (GCT) in a practical way might be valuable for many runners and coaches.

The Myotest Run is a practical 3D accelerometer that has been developed as a field-based running device meant to be used outside such as on an athletic track by individual runners and coaches (Myotest, 2012). The Myotest allows to record, process, display and store data related to running economy and performance. Specifically, the Myotest provides data on variables related to running technique such as SF, stride length, vertical oscillation, GCT and reactivity. Previous studies conducted in laboratory setting on a treadmill have shown some favorable findings towards the measurement quality of this field-based running device (high reproducibility) (Bampouras et al., 2010; Nuzzo et al., 2011). Whether the measurement quality of the Myotest for the assessment of important running-related technique aspects such as SF and GCT is also favorable in a more practical setting such as an athletic track remains unknown.

The measurement quality of any instrument, test or device, specifically referring to reproducibility and validity, needs to be explored before its use in practice (de Vet et al., 2011). An instrument is considered reproducible if its measurements are consistent and stable over time from one test moment to another (free from significant random error), under the assumption that the characteristic being measured does not change over time (de Vet et al., 2011). Reproducibility relates to two concepts, namely reliability and agreement (de Vet et al., 2011). Reliability refers to an instrument’s ability to distinguish one subject from another despite measurement errors, while agreement concerns the absolute measurement error, evaluating how close the scores are in repeated measurements (de Vet et al., 2011). An instrument is considered valid when it measures what it intends to measure (free from significant systematic error) (de Vet et al., 2011). Concurrent validity, an important aspect of validity, examines at the same time how the evaluated instrument relates to an existing, highly valued instrument called a gold standard (shown to be reproducible and valid) that measures the same parameter or concept (de Vet et al., 2011).

According to the aforementioned considerations, we aimed to explore the measurement quality of the Myotest in terms of reproducibility and validity, using a foot-mounted accelerometer as gold standard as it has been shown valid to measure SF and GCT. Our research questions were twofold: what is the reproducibility (test-retest reliability and agreement) of the Myotest for measuring SF and GCT in recreational runners and what is the concurrent validity of the Myotest with foot-mounted accelerometers for measuring SF and GCT in recreational runners?

## Material and Methods

### Participants

Participants were healthy recreational runners, recruited at a running association in Amsterdam. To be eligible to be enrolled in our study, participants were required to meet the following inclusion criteria: (1) free from any running-related musculoskeletal injury in the past month, (2) being weekly active in running during the past month, and (3) being 18 years old or older. Sample size calculation (nQuery Advisor: confidence interval [CI] method with a confidence level of 0.95, correlation coefficient set at 0.90 and limit at 0.70) indicated that at least 14 subjects were required for this study. Consequently, 14 recreational runners (11 men, 3 women) participated in our study. Their mean age was 45 ±14 years (range, 20–68 yrs), mean height was 181 ±7cm (range, 165–188 cm), and mean body weight was 77 ±11kg (range 53–90 kg). Prior to enrollment, and after receiving verbal and written information on the study aim and procedures, participants signed statements of informed consent. Subjects were free to quit the study at any time.

### Myotest

The Myotest is a small device (W × L × H: 54.2 × 102.5 × 10.7 mm, weight 59 g, sample frequency 200–500 Hz) attached with a Velcro waistband to the runner (Moytest, 2012). The Myotest Runcheck software provides several running-related parameters among which SF (in steps per minute) and GCT (in milliseconds). Once set up in accordance to a runner’s characteristics (sex, height, weight and level of expertise), the device was attached to the Velcro waistband around the runner’s iliac crest, on the ventral side of the body. This standardized position allows the runner to keep their full range of motion. Then, the runner only had to press the enter button in the middle of the device to start the data collection. The same enter button needed to be pressed to stop the device. For our study, the level of expertise was set to “Expert” for all subjects.

### Gold standard

**Figure 1:**
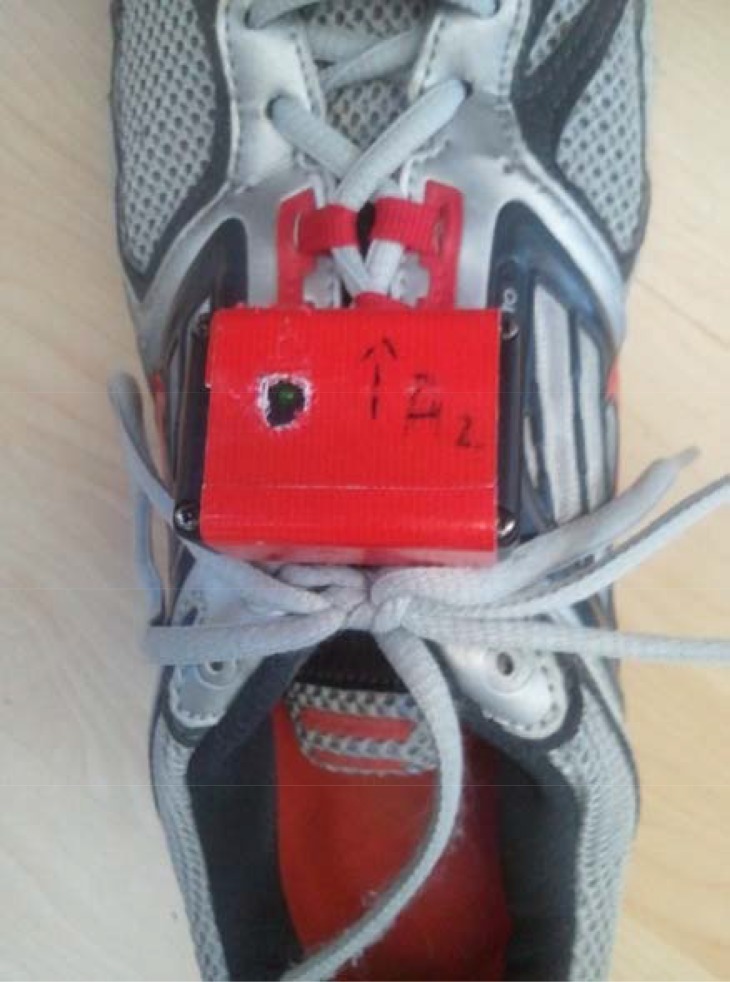
Foot-mounted accelerometer (Gold Standard) attached to the runner’s shoe

Foot-mounted accelerometer was used in our study as gold standard, as it has been developed and validated to measure SF and GCT (de Ruiter et al., 2013). Containing a tri-axial accelerometer (±6 g; 1000 Hz, MMA7361L, Freescale Semiconductor, Austin, Texas, USA), the foot-mounted accelerometer uses a software algorithm (MATLAB R2010a, Mathworks, Natick, USA) based on the open-source platform Arduino. A foot-mounted accelerometer was attached at each shoe of the participant by using the shoe lace, sports tape being also used to secure its sustainable position (Figure 1). Transmitting data wirelessly, SF (in steps per minute) and GCT (in milliseconds) were calculated automatically and exported directly to a Microsoft Excel file.

### Procedures

An experimental study using a within-subjects design (test-retest) was conducted to assess reproducibility and concurrent validity of the Myotest. Each participant was assessed during two test sessions, using a time interval of 7 ±4 days between both test days. We assumed that such a time interval was optimal to assure a steady state in participants. In addition, participants were asked to wear the same running shoes during both test sessions. Participants were asked before each test session to avoid any training session and exhaustive event in the previous 24 and 36 hours, respectively. Prior to each test session, measurement devices (Myotest and foot-mounted accelerometer) were attached to the participants by the same researcher (NG) and set up in order to be ready for measurement. Before each test session, participants were informed one more time about the experimental procedures in order to prevent misunderstanding, and were asked to perform a standardized warm-up (jogging at a comfortable pace without fatigue development). A test session consisted of three runs of 400 meters on an outdoor athletic track: the first run at an approximated speed of 10 km/h, the second run at an approximated speed of 12 km/h, and the third run at an approximated speed of 14 km/h (approximated speed fed back verbally every 100 m). Between the three runs, participants were allowed to rest as required but up to 2 minutes. During the first test session, SF and GCT were assessed concurrently by the Myotest and the foot-mounted accelerometer (gold standard). During the second test session, SF and GCT were measured only by the Myotest. Ethical approval was not needed from the ethical committee of the Faculty of Human Movement Sciences of the VU University as the study did not fall into the Medical Research Involving Human Subjects Act. The study was carried out in accordance with the Declaration of Helsinki (2000).

### Statistical Analysis

Means, standard deviations (SD’s), and ranges were calculated for each outcome measure at each test session. Reliability and agreement were determined using the SF and GCT outcomes assessed by the Myotest during the two test sessions (test and retest). The level of test-retest reliability was expressed with the intraclass correlation coefficient (ICC; two-way random model, agreement, single measures) and its 95% confidence interval (95% CI) (Portney and Watkins, 2008; de Vet et al., 2011). Agreement was expressed with the standard error of measurement (SEM = √ [var(raters) + var(error)] or SEM = SD x √[1 – ICC]). Concurrent validity was investigated by comparing the SF and GCT outcomes assessed by the Myotest to the SF and GCT outcomes assessed by the foot-mounted accelerometer (gold standard) (Portney and Watkins, 2008; de Vet et al., 2011). The level of concurrent validity was expressed with the intraclass correlation coefficient (ICC; two-way random model, consistency, single measures) and its 95% confidence interval (95% CI) (Portney and Watkins, 2008; de Vet et al., 2011). ICC’s obtained for reliability and concurrent validity were interpreted as good for ICC > 0.75, as moderate for 0.50 ≤ ICC ≤ 0.75, and as poor for ICC < 0.50 (Portney and Watkins, 2008; de Vet et al., 2011). All data analyses were performed using the statistical software IBM SPSS Statistics 22.0 for Windows.

## Results

### Reliability and agreement

Table 1 presents the averages, SD’s, and ranges of the SF and GCT measured with the Myotest at different speeds during both test sessions (test and retest), and their related ICC’s (95% confidence interval) and SEM’s. The level of test-retest reliability of the Myotest for SF was good at all three running speeds, with ICC’s ranging from 0.78 to 0.92. The SEM of the Myotest for SF, expressed in steps per minute, were rather small given the mean values found during both test sessions. For instance, mean SF measured by the Myotest at 14 km/h was 175–176 steps per minute and its SEM 3 steps per minute, indicating that an increase or decrease of 6 steps per minute cannot be interpreted as a random measurement error. The level of test-retest reliability of the Myotest for GCT was poor to moderate at different running speeds as ICC’s ranged from −0.24 to 0.67. The SEM of the Myotest for GCT, expressed in ms, were not acceptable, being large relative to the mean values found during both test sessions. For instance, mean GCT measured by the Myotest at 12 km/h was 156–159 ms and its SEM 15 ms, indicating that an increase or decrease of more than 30 ms needs to be reached before one can interpret it as more than a random measurement error.

### Concurrent validity

Table 2 presents the averages, SD’s, and ranges of the SF and GCT measured with the Myotest and foot-mounted accelerometer (gold standard) at different speeds during the first test session, and their related ICC’s (95% confidence interval). The level of concurrent validity of the Myotest with the foot-mounted accelerometer (gold standard) for SF was good at all three running speeds as ICC’s ranged from 0.78 to 0.90. The level of concurrent validity of the Myotest with the foot-mounted accelerometer (gold standard) for GCT was only moderate at all three running speeds as ICC’s ranged from 0.48 to 0.50.

## Discussion

Using a within-subjects design on runners free from musculoskeletal injuries, the purpose of our study was to evaluate the reliability, agreement and concurrent validity of the Myotest for measuring SF and GCT at three different running speeds. For SF, test-retest reliability and agreement of the Myotest were evaluated as good at all running speeds. For GCT, test-retest reliability was found to be moderate at a running speed of 14 km/h and poor at speeds of 10 and 12 km/h. Agreement of the Myotest for GCT at all three running speeds was not acceptable given the large SEM. Concurrent validity of the Myotest with the foot-mounted accelerometer (gold standard) at all three running speeds was found to be good for SF and moderate for GCT.

Empirical studies assessing the measurement quality of the Myotest are scarce, especially related to the assessment of running parameters. In a previous study, reliability, agreement and validity of the Myotest for measuring running economy and vertical oscillation were explored among healthy runners (Potter et al., submitted). For both running economy and vertical oscillation, levels of (test-retest) reliability were moderate to good (ICC > 0.50) while SEM (agreement) were acceptable relative to the mean values (Potter et al., submitted). In contrast, the validity of the Myotest for measuring running economy and vertical oscillation was only poor to moderate (Potter et al., submitted). For measuring other performance parameters such as force (countermovement vertical jump), the Myotest was found to be highly reliable (test-retest reliability) and valid, endorsing its application in the field for the assessment of force related parameters (Bampouras et al., 2010; Nuzzo et al., 2011). Other accelerometers similar to the Myotest have also shown some moderate to high evidence of test-retest reliability and validity (Thompson and Bemben, 1999; Brage et al., 2003; Esliger et al., 2006). However, most of these studies were conducted in laboratory setting and not specifically related to running parameters.

Our study was conducted in a practical context, which can be seen as a strength of our experiment. The Myotest is a small and practical 3D accelerometer that has been developed as a field-based running device meant to be used outside such as on an athletic track by individual runners and coaches (Myotest, 2012). Consequently, even though the use of a treadmill might offer safer and more controlled conditions, the measurement quality of the Myotest should in principle be determined for measurements in the field such as on an athletic track. A gait on a treadmill has been shown to differ from a gait on an outside track, endorsing the choice for our experimental conditions (Kirtley, 2006). When it comes to criterion-related validity (concurrent and predictive), the availability of a gold standard, an instrument already known as reliable and valid measuring the same construct, is crucial (de Vet et al., 2011). In our study, we chose a foot-mounted accelerometer, as it has been validated for SF and GCT by comparing it to a force platform (de Ruiter and al., 2013).

With regard to our findings, the Myotest was found to be reproducible and valid for measuring SF at 10, 12 and 14 km/h. It has been shown that SF between 180 and 185 steps per minute significantly decreases biomechanical loads on the lower extremities (peak vertical ground reaction force, energy generated by the hip, knee and ankle joints, ground reaction force and compartment pressures), compared to lower SF usually preferred by novice runners (Boschman and Gouttebarge, 2013). Also running economy can be improved in novice runners by adopting a higher SF (de Ruiter et al., 2013). Consequently, runners (or their coaches) could use the Myotest to get feedback about their current SF, striving to learn to run at higher SF and using the Myotest to monitor this process over time. By contrast to SF, since no instrument can be valid if not reliable (de Vet et al., 2011), the Myotest was not reliable nor valid for measuring GCT and thus cannot be used for measuring GCT. In addition, the GCTs found with the myotest (Table 2) are too low when compared with values (250–350 ms) reported in the literature at these speeds (de Ruiter et al., 2013).

In conclusion, our study results suggest that the Myotest is a practical, useful, reliable and valid device that can be used by runners and coaches to assess and monitor SF outside on an athletic track. For measuring GCT, the Myotest should not be used yet as it was not sufficiently reliable nor valid. Future research focusing on the Myotests’s reproducibility and validity for measuring other running technique elements, such as vertical oscillation, stride length or foot strike pattern, when running on an athletic track is needed. In addition, attention should also be given to responsiveness when change in running technique elements over time is aimed for.

## Figures and Tables

**Table 1:** Mean scores, standard deviation and range obtained from the Myotest for step frequency (SF; step per minute) and ground contact time (GCT; ms) at different running speed, and the level of test-retest reliability and agreement of the Myotest

	Test session 1			Test session 2			ICC	(95% CI)	SEM
	
	Mean	SD	Range	Mean	SD	Range			
SF at 10 km/h	164.3	7	155–181	164.4	9	153–183	0.82	0.52–0.94	3.5
SF at 12 km/h	168.9	8	161–185	169.4	10	161–193	0.78	0.44–0.92	4.1
SF at 14 km/h	175.9	10	163–199	176.9	10	164–203	0.92	0.77–0.97	3.0

GCT at 10 km/h	172.0	15	154–204	165.4	17	113–188	−0.24	−0.69–0.32	n/a
GCT at 12 km/h	159.1	17	123–194	156.4	20	106–189	0.35	−0.23–0.74	14.8
GCT at 14 km/h	144.2	16	103–177	142.9	18	102–176	0.67	0.22–0.88	10.1

SD, standard deviation; ICC, Intra-Class correlation coefficient; CI, confidence interval; SEM, standard error of measurement; n/a, not applicable

**Table 2:** Mean scores, standard deviation and range obtained from the Myotest and foot-mounted accelerometer (Gold Standard) for step frequency (SF; step per minute) and ground contact time (GCT; ms) at different running speed, and the level of concurrent validity

	Myotest			foot-mounted accelerometer			ICC	(95% CI)
	
	Mean	SD	Range	Mean	SD	Range		
SF at 10 km/h	164.3	7	155–181	165.6	8	156–183	0.89	0.69–0.96
SF at 12 km/h	168.9	8	161–185	169.4	8	161–184	0.78	0.45–0.96
SF at 14 km/h	175.9	10	163–199	175.7	13	157–198	0.90	0.72–0.97

GCT at 10 km/h	172.0	15	154–204	297.1	20	256–331	0.49	−0.03–0.80
GCT at 12 km/h	159.1	17	123–194	278.4	25	241–314	0.50	−0.02–0.81
GCT at 14 km/h	144.2	16	103–177	251.3	24	205–274	0.48	−0.07–0.81

SD, standard deviation; ICC, Intra-Class correlation coefficient; CI, confidence interval; n/a, not applicable
